# Deconstructing the Alcohol Harm Paradox: A Population Based Survey of Adults in England

**DOI:** 10.1371/journal.pone.0160666

**Published:** 2016-09-28

**Authors:** Emma Beard, Jamie Brown, Robert West, Colin Angus, Alan Brennan, John Holmes, Eileen Kaner, Petra Meier, Susan Michie

**Affiliations:** 1 Research Department of Clinical, Educational and Health Psychology, University College London, London, United Kingdom; 2 Department of Epidemiology and Public Health, University College London, London, United Kingdom; 3 ScHARR, School of Health and Related Research, University of Sheffield, Sheffield, United Kingdom; 4 Institute of Health & Society, Newcastle University, Newcastle, United Kingdom; National Institute of Health, ITALY

## Abstract

**Background:**

The Alcohol Harm Paradox refers to observations that lower socioeconomic status (SES) groups consume less alcohol but experience more alcohol-related problems. However, SES is a complex concept and its observed relationship to social problems often depends on how it is measured and the demographic groups studied. Thus this study assessed socioeconomic patterning of alcohol consumption and related harm using multiple measures of SES and examined moderation of this patterning by gender and age.

**Method:**

Data were used from the Alcohol Toolkit Study between March and September 2015 on 31,878 adults (16+) living in England. Participants completed the AUDIT which includes alcohol consumption, harm and dependence modules. SES was measured via qualifications, employment, home and car ownership, income and social-grade, plus a composite of these measures. The composite score was coded such that higher scores reflected greater social-disadvantage.

**Results:**

We observed the Alcohol Harm Paradox for the composite SES measure, with a linear negative relationship between SES and AUDIT-Consumption scores (β = -0.036, p<0.001) and a positive relationship between lower SES and AUDIT-Harm (β = 0.022, p<0.001) and AUDIT-Dependence (β = 0.024, p<0.001) scores. Individual measures of SES displayed different, and non-linear, relationships with AUDIT modules. For example, social-grade and income had a u-shaped relationship with AUDIT-Consumption scores while education had an inverse u-shaped relationship. Almost all measures displayed an exponential relationship with AUDIT-Dependence and AUDIT-Harm scores. We identified moderating effects from age and gender, with AUDIT-Dependence scores increasing more steeply with lower SES in men and both AUDIT-Harm and AUDIT-Dependence scores increasing more steeply with lower SES in younger age groups.

**Conclusion:**

Different SES measures appear to influence whether the Alcohol Harm Paradox is observed as a linear trend across SES groups or a phenomenon associated particularly with the most disadvantaged. The paradox also appears more concentrated in men and younger age groups.

## Introduction

Around 9.1 million adults in England drink alcohol above recommended limits, which leads to a wide range of health and social issues, from dangerous driving to crime, cancer and domestic abuse [1, 2]. The 2014 World Health Organisation’s global status report estimated that 5.9% of all global deaths and 5.1% of the global burden of disease and injury is attributable to alcohol each year [3]. Despite those of higher SES being more likely to report engagement in hazardous levels of drinking, these health and social issues disproportionally affect the most deprived communities [4–19]. This phenomenon, whereby alcohol consumption tends to be higher in people of higher socio-economic status (SES), while levels of alcohol-related problems are greater in people of lower SES, has been coined the Alcohol Harm Paradox [4, 5]. Understanding what underlies this phenomenon requires more information on whether it is observable across different markers of SES and if it is moderated by other demographic variables including age and gender [4, 5, 20].

Elucidating the association between different SES measures and alcohol harm may help to identify possible causes of the Alcohol Harm Paradox by pinning down its underlying mechanisms. At the same time, the identification of measures by which the Alcohol Harm Paradox consistently emerges will be of interest to those planning studies on the association between SES and alcohol-related problems. Although it is important to choose a valid and reliable measure of SES, it is equally important to select one which captures the phenomenon of interest. One way researchers have addressed this issue is by use of composite scores which have been argued to reflect the multifaceted nature of SES i.e. both the human, social and material capital aspects [21, 22]. However, due to costs and logistical constraints it is often not possible to use a wide range of measures, and so it is important to form a consensus on which are the optimal choices [23]. It is also important to determine whether the Alcohol Harm Paradox differs as a function of socio-demographics, with previous studies hinting towards the Alcohol Harm Paradox being more pronounced among younger adults [19, 24–28]and men [29]. This will help target interventions and policies aimed at driving down inequalities in health.

The Alcohol Use Disorders Identification Test (AUDIT) [30] affords the ability to address these issues. The AUDIT combines scores on self-reported answers to questions regarding injuries to oneself or others; the need to drink first thing in the morning; feelings of remorse and guilt; an inability to stop drinking; concerns raised by friends, family and health-care professions; being unable to do what one would normally do; and episodes of alcohol induced amnesia. The AUDIT also includes a consumption measure which assesses frequency and quantity of usual drinking, as well as frequency of ‘binge’ drinking. Previous studies have found that overall scores on the AUDIT are higher among those of lower SES, even though consumption scores tend to be lower or the same; indicating more harmful, hazardous, and potentially dependent, levels of drinking [29, 31–33].

Although objective measures of harm are available (e.g., alcohol-related mortality, morbidities and hospital admissions), these have a tendency to underestimate the true impact. For example, alcohol-related deaths only apply to those for which alcohol was the main contributor [34]. Other issues arise in terms of attributing admissions and illnesses to alcohol and in attaching such measures to an individual of a particular SES. The AUDIT also benefits from allowing a wider assessment of harms that are less amendable to objective measurement [9], which in themselves have been found to be predictive of future acute events and offer an opportunity for early intervention [35, 36].

In summary, this paper aimed to:

1. Identify the Alcohol Harm Paradox in a population sample of adults in England by assessing the association between a composite measure of SES and: a) Drinking status and b) AUDIT measures: i) Alcohol consumption (AUDIT-C), ii) Alcohol-related harm (AUDIT-Harm) and iii) an Alcohol-dependence indicator (AUDIT-Dependence).
2. Assess whether the Alcohol Harm Paradox exists across different measures of SES, and if the pattern of association is similar for these individual measures (i.e. linear or non-linear), by assessing the association between social-grade, housing tenure, car ownership, qualifications, income and employment status and: a) Drinking status and b) AUDIT measures: AUDIT-C, ii) AUDIT-Harm and iii) AUDIT-Dependence.
3. Assess whether the Alcohol Harm Paradox is moderated by demographic characteristics by assessing the association between an interaction of the composite score of SES with gender and age and AUDIT measures: i) AUDIT measures: AUDIT-C, ii) AUDIT-Harm and iii) AUDIT-Dependence.

All analyses were adjusted for gender, age, ethnicity and alcohol consumption, given that alcohol use varies as a function of demographic characteristics and as differences in drinking patterns may be one causal explanation for the Alcohol Harm Paradox [5, 37]. This is the first study, to our knowledge, which has investigated the Alcohol Harm Paradox in a large population sample of adults in England using multiple measures of SES, and its moderation by demographic characteristics.

## Methodology

### Ethical approval

Ethics approval for the Smoking Toolkit Study (STS), a sister survey to the Alcohol Toolkit Study (ATS), was originally granted by the UCL Ethics Committee (ID 0498/001). Approval for the ATS was granted by the same committee as an extension of the STS. The data are not collected by UCL and are anonymised when received by UCL. Explicit verbal agreement and willingness to answer questions voluntarily is recorded electrically by Ipsos Mori, the company administering the survey. This is standard protocol and was agreed by the UCL ethics committee. Participants are also given a printed information sheet.

### Design

Data were used from the ATS (www.alcoholinengland.info). The sample comprised of all those taking part between March 2014 and September 2015 (the period for which data were available). The ATS involves monthly cross-sectional household computer-assisted interviews, conducted by Ipsos Mori of approximately 1,700 adults aged 16+ and over in England. The baseline survey uses a type of random location sampling, which is a hybrid between random probability and simple quota sampling [38]. Participants from the STS appear to be representative of the population in England, having similar socio-demographic composition and smoking characteristics to large national surveys based on probability samples such as the Health Survey for England [17]. STROBE reporting guidelines were followed throughout [39].

### Measures

Data were collected on participant’s age, gender, ethnicity and SES. Six measures of SES were collected which are outlined below.

1. Social-grade was measured using the British National Readership Survey (NRS) Social-Grade Classification Tool [40].
2. Annual income in 15 bands (Up to £4499; £4,500–6,499; £6500–7499; £7500-£9499; £9500–11499; £11500-£13499; £13500–15499; £15500–17499; £17500–24999; £25000–29999; £30000–39999; £40000–49999; £50000–74999; £75000-£99999; > £100000).
3. Educational level in 8 categories (GCSE/O-level/CSE; vocational qualification; A-level or equivalent; Bachelor degree or equivalent; Masters/PHD or equivalent; other; no formal qualifications; still studying)
4. Car ownership (owns a car; does not own a car)
5. Working status in 7 categories (Have paid job (full time); have a paid job (part time and over or under 8 hours per week); self-employed; full-time student; still at school; retired; not in paid work (long term illness, housewife or other reason)
6. Housing tenure in 6 categories (mortgage, owned outright, rented from local authority, rented from private landlord, belongs to housing association and other).

All variables, except social-grade, were then dichotomised or categorised as follows (all variables were coded so that lower SES reflected higher scores): 1) Income: £40,000 +, £17,500 to £39,999, £11,500 to £17,499, < £11,499, per annum; 2) Education: University education, A-level and equivalent, GCSE/vocational, other/still studying, none; 3) Car ownership: Owns a car versus does not own a car; 4) Working status: Full time job versus no full time job; and 5) Housing tenure: owner occupied (owned outright or being brought with a mortgage) versus other. These thresholds were based on previous research and characterisations: income was categorised into quartiles, with the cut-off of £11,499 being the closest equivalent to the UK definition of poverty of 60% of median national household income [41]. Educational categorises, home ownership and full time employment have also been previously used (e.g., [42, 43].

A composite score was also derived to reflect the multidimensional nature of SES. Composite scores have the advantage of reducing measurement error present in single items and improving ease of reporting and interpretation [21, 44]. The composite score was coded such that a higher composite score measure reflected greater social-disadvantage.

Finally, participants completed the 10-item AUDIT [45]. Questions 1–3 deal with alcohol consumption, 4–6 with alcohol dependence and 7–10 with alcohol-related harm. Previous research suggests these three AUDIT dimensions provide sensitive and coherent measures of alcohol consumption, harm and dependence [45, 46].

### Analysis

All analyses were conducted in R version 3.1.2. Percentages of missing values for the predictor variables of interest were as follows: gender = 0%, age = 0%, social-grade = 0%, ethnicity = 0.5%, home ownership = 1.0%, full time work = 0.3%, income = 42.8%, car ownership = 1.2%. Missing data for the 10 items of the AUDIT ranged from 0.1 to 0.8%. Missing data were imputed by multiple imputation using the Amelia 11 package [47]. Little’s test suggested that income data may not have been missing at random [48]. The number of imputed data sets was set to 20 [49] and results combined using Rubin’s Rules [50].

A SES composite score, based on all 6 measures of SES, was derived using Multiple Correspondence Analysis (MCA) applied using the FactoMineR package [51]. Weights for the composite score comprised of those for the first three components; the assumption being that the variation explained by these is sufficient to adequately represent the original values [52].

The analysis proceeded as follows:

1. Sample characteristics
  Data were weighted for important prevalence statistics using the “Survey” R package [53], in order to match the population in England. Generalised Linear Models (GLM), specifying the quasi-binomial distribution, were used to assess the association between socio-demographic characteristics and abstinence/alcohol use.
2. Identifying the Alcohol Harm Paradox
  Separate GLMs, specifying the Gaussian family, were run to assess the associations between the composite measure and AUDIT dimension scores. Associations with AUDIT-C, AUDIT-Harm and AUDIT-Dependence were only assessed among those who reported alcohol consumption. All analyses were adjusted for age, gender and ethnicity (and alcohol consumption when assessing AUDIT-Harm and AUDIT-Dependence). The associations between the SES measures and each of its 10 component scales were also assessed in sensitivity analyses (see S1 Table).
3. Does the Alcohol Harm Paradox exist across different measures of SES?
  In order to assess whether the composite score suitably reflected the associations between the individual SES measures and the outcomes of interest, additional models were run which regressed the 6 individual SES measures onto the residuals of the fully adjusted models. Lack of association would suggested that the residual variance is simply noise, while significant relationships would suggest that the individual items may explain additional patterns in AUDIT dimension scores and drinking status not captured by the composite measure. Further GLMs were then run to assess the association between these individual measures and the three AUDIT dimension scores.
4. Is the Alcohol Harm Paradox moderated by demographic characteristics?
  Possible moderation effects of gender and age were assessed by including interaction terms in the GLM models. Moderation effects were only assessed for the composite score due to ease of reporting, as it aims to reflect the multifaceted nature of SES, had the most consistent relationships with the ten questions of the AUDIT (see S1 Table), and is less susceptible to measurement error [21].

For all analyses, age was categorised as the assumptions of linearity and linearity of the logit were violated.

## Results

### Sample characteristics

Data were collected on 31,878 participants between March 2014 and September 2015. Sixty-eight per cent of participants reported that they drank alcohol (95%CI 67.0 to 68.1; n = 21539; Weighted = 70.7, 95%CI 70.2 to 71.2, n = 22538). Table 1 shows the demographic characteristics of participants overall and as a function of their drinking status. Women and those of non-white ethnicity had lower odds of consuming alcohol; while those of an older age were more likely to report that they drank than those aged 16–24.

**Table 1:** Demographic characteristics overall and as a function of drinking status (Drinker versus Non-Drinker).

	All participants (n = 31,878)	Non-Drinkers (n = 10339)	Drinkers (n = 21539)	OR (95%CI) unadjusted
**Gender %(n)**				
*Male*	51.1 (16279)	45.9 (4748)	53.5 (11531)	Reference
*Female*	48.9 (15599)	54.1 (5591)	46.5 (10008)	0.74 (0.70 to 0.77)***
**Age %(n)**				
*16–24*	16.0 (5092)	18.2 (1879)	14.9 (3113)	Reference
*25–34*	15.4 (4917)	19.2 (1983)	13.6 (2934)	0.87 (0.80 to 0.94)***
*35–44*	14.6 (4646)	15.9 (1647)	13.9 (2999)	1.06 (0.98 to 1.16)
*45–54*	15.1 (4816)	12.7 (1315)	16.3 (3501)	1.56 (1.43 to 1.70)***
*55–64*	15.0 (4792)	12.1 (1249)	16.4 (3543)	1.66 (1.52 to 1.81)***
*65+*	23.9 (7615)	21.9 (2266)	24.8 (5349)	1.38 (1.28 to 1.49)***
**Ethnicity %(n)**				
*White*	83.2 (26028)	61.3 (6344)	92.9 (20001)	Reference
*Non-white*	16.8 (5333)	38.7 (4008)	7.07 (1525)	0.12 (0.11 to 0.13)***

Note:

*** significant difference *p*<0.001.

### Identifying the Alcohol Harm Paradox

The composite score was derived using the weights from the MCA given in Table 2. The derived composite score was found to have good internal consistency (standardised Cronbach alpha of: 0.64). Reliability decreased when SES measures were dropped, with the greatest decline being the exclusion of social-grade (Cronbach alpha to 0.53).

**Table 2:** ETA squared values (weights) for the dimensions of the composite score.

Dimension	Eta squared
Home ownership	0.406
Full time work	0.453
Income	0.878
+16 Education	1.167
Car ownership	0.186
Social-grade	1.631

Table 3 shows the results of the linear regression analyses assessing the association between the composite score with drinking status and the three AUDIT dimensions. Those with higher composite scores had lower odds of drinking alcohol compared to those with lower composite scores (non-drinkers composite score: M = 12.5, SD±3.93; drinkers composite score: M = 10.5, SD± 3.72). In the fully adjusted models, increasing social-disadvantage was positively associated with both AUDIT-Harm and AUDIT-Dependence scores.

**Table 3:** Association between the composite SES score with drinking status and the three dimensions of the AUDIT.

	Adjustment	Drinkers versus non-drinkers	AUDIT-C	AUDIT-Harm	AUDIT-Dependence
		OR (95%CI) *p*	β (95%CI) *p*	β (95%CI) *p*	β (95%CI) *p*
***Model 1***	None	0.873 (0.867 to 0.879) 0.001	-0.050 (-0.059 to -0.041) 0.001	0.011 (0.005 to 0.016) 0.001	0.018 (0.015 to 0.021) 0.001
***Model 2***	*Gender*, *age & ethnicity*	0.862 (0.856 to 0.869) 0.001	-0.036 (-0.045 to -0.027) 0.001	0.013 (0.008 to 0.019) 0.001	0.020 (0.017 to 0.023) 0.001
***Model 3***	+ AUDIT-C			0.022 (0.017 to 0.027) 0.001	0.024 (0.021 to 0.027) 0.001

Note: NA = Not applicable; composite score was mean centred

### Does the Alcohol Harm Paradox exist across different measures of SES?

The results of the residual analyses, whereby the 6 individual SES measures were regressed onto the residuals of the fully adjusted models in Table 3, are given in Table 4. Although it appears that the majority of residual variance may be noise for drinking status and AUDIT-C, significant relationships between some of the SES measures and residuals, particularly education and social-grade, were found for AUDIT-Dependence and AUDIT-Harm. This suggests that some non-linear associations between the various SES measures and AUDIT-Dimensions may exist.

**Table 4:** Association between the individual SES measures and residuals of the adjusted models assessing the association between the composite score with drinking and the three AUDIT dimensions with adjustment for composite scores.

	Drinkers versus non-drinkers	AUDIT-C	AUDIT-Harm	AUDIT-Dependence
	β (95%CI)	β (95%CI)	β (95%CI)	β (95%CI)
**Social-grade %(n)**				
*AB*	Reference	Reference	Reference	Reference
*C1*	0.09 (<0.01 to 0.18)	-0.09 (-0.19 to 0.01)	-0.03 (-0.09 to 0.02)	-0.04 (-0.07 to <0.01)*
*C2*	0.15 (0.03 to 0.27) *	-0.10 (-0.23 to 0.03)	-0.15 (-0.22 to -0.08)***	-0.05 (-0.10 to <-0.01)*
*D*	0.05 (-0.01 to 0.21)	-0.26 (-0.43 to -0.08)**	-0.17 (-0.26 to -0.07)**	-0.10 (-0.16 to -0.04)**
*E*	0.26 (0.07 to 0.45) **	0.18 (-0.04 to 0.40)	0.03 (-0.10 to 0.15)	0.08 (<-0.01 to 0.15)
**Home ownership %(n)**				
*Yes*	Reference	Reference	Reference	Reference
*No*	-0.01 (-0.17 to -0.04) ***	0.17 (0.1 to 0.24)***	0.12 (0.08 to 0.16)***	0.07 (0.05 to 0.10)***
**Income %(n)**				
*£40*,*000 +*	Reference	Reference	Reference	Reference
*£17*,*500 to £39*,*999*	-0.02 (-0.11 to 0.06)	-0.22 (-0.32 to -0.12)***	0.04 (-0.01 to 0.09)	-0.01 (-0.04 to 0.02)
*£9*,*500 to £17*,*499*	-0.13 (-0.26 to <0.01)	-0.38 (-0.50 to -0.26)***	0.09 (0.02 to 0.15)*	0.02 (-0.03 to 0.07)
*< £11*,*499*	0.01 (-0.12 to 0.14)	-0.01 (-0.14 to 0.13)	0.28 (0.19 to 0.36)***	0.09 (0.04 to 0.14)***
**Car ownership %(n)**				
*Yes*	Reference	Reference	Reference	Reference
*No*	-0.01 (-0.07 to 0.05)	(<0.01 (-0.06 to 0.07)	0.04 (<0.01 to 0.08)*	0.05 (0.03 to 0.07)***
**Education %(n)**				
*University*	Reference	Reference	Reference	Reference
*A-level and equivalent*	0.14 (0.05 to 0.23) **	0.27 (0.17 to 0.37)***	-0.09 (-0.14 to -0.03)**	-0.05 (-0.08 to -0.01)***
*GCSE/vocational*	0.02 (-0.08 to 0.11)	0.08 (-0.03 to 0.18)	-0.16 (-0.22 to -0.01)***	-0.09 (-0.13 to -0.06)***
*Other/still studying*	0.14 (0.01 to 0.27) *	0.02 (-0.12 to 0.16)	-0.24 (-0.32 to -0.16)***	-0.12 (-0.17 to -0.07)***
*None*	-0.13 (-0.26 to 0.01)	<0.01 (-0.16 to 0.16)	-0.25 (-0.34 to -0.17)***	-0.12 (-0.18 to -0.07)***
**Full time work %(n)**				
*Yes*	Reference	Reference	Reference	Reference
*No*	0.10 (0.04 to 0.16) ***	0.24 (0.17 to 0.30)***	0.13 (0.09 to 0.17)***	0.06 (0.03 to 0.08)***

Note:

*significant difference *p*<0.05;

** significant difference *p*<0.01;

*** significant difference *p*<0.001

Table 5 shows the association between the individual measures of SES and drinking status (i.e. drinker versus non-drinker). In the adjusted analyses, the odds of being a drinker decreased linearly with decreasing social-grade, income and educational attainments. Those who did not own a car, did not own their own home, and were not in full time work, also had lower odds of reporting that they drank alcohol.

**Table 5:** Socio-demographic characteristics and AUDIT dimension scores overall and as a function of drinking status (Drinker versus Non-Drinker).

	All participants (n = 31,878)	Non-Drinkers (n = 10339)	Drinkers (n = 21539)	OR (95%CI) unadjusted	OR (95%CI) adjusted for gender, age and ethnicity
**Social-grade %(n)**					
*AB*	21.0 (6692)	12.3 (1275)	25.1 (5417)	Reference	Reference
*C1*	30.7 (9798)	26.2 (2706)	32.9 (7092)	0.62 (0.57 to 0.66)***	0.68 (0.63 to 0.74)***
*C2*	21.0 (6704)	20.7 (2145)	21.2 (4559)	0.50 (0.46 to 0.54)***	0.49 (0.45 to 0.54)***
*D*	16.0 (5112)	23.3 (2404)	12.6 (2708)	0.27 (0.24 to 0.29)***	0.30 (0.27 to 0.33)***
*E*	11.2 (3572)	17.5 (1809)	8.2 (1763)	0.23 (0.21 to 0.25)***	0.24 (0.22 to 0.27)***
**Home ownership %(n)**					
*Yes*	59.7 (18502)	49.1 (5082)	65.6 (14126)	Reference	Reference
*No*	40.3 (12300)	50.9 (5270)	34.4 (7400)	0.51 (0.48 to 0.53)***	0.54 (0.51 to 0.57)***
**Income %(n)**					
*£40*,*000 +*	25.7 (8007)	18.2 (1881)	29.7 (6449)	Reference	Reference
*£17*,*500 to £39*,*999*	31.9 (9862)	28.6 (2959)	33.8 (7265)	0.72 (0.66 to 0.78)***	0.74 (0.68 to 0.80)***
*£9*,*500 to £17*,*499*	20.8 (6365)	25.3 (2619)	18.3 (3942)	0.44 (0.40 to 0.48)***	0.48 (0.43 to 0.53)***
*< £11*,*499*	21.6 (6569)	28.0 (2896)	18.0 (3868)	0.39 (0.36 to 0.43)***	0.41 (0.38 to 0.45)***
**Car ownership %(n)**					
*Yes*	33.1 (10567)	28.0 (2903)	35.6 (7663)	Reference	Reference
*No*	66.9 (21312)	72.0 (7449)	64.4 (13863)	0.71 (0.67 to 0.74)***	0.79 (0.74 to 0.83)***
**Education %(n)**					
*University*	27.4 (8723)	22.0 (2280)	29.9 (6443)	Reference	Reference
*A-level and equivalent*	18.1 (5778)	15.4 (1593)	19.4 (4185)	0.93 (0.86 to 1.00)	0.81 (0.75 to 0.89)***
*GCSE/vocational*	28.4 (9047)	28.5 (2955)	28.3 (6093)	0.73 (0.68 to 0.78)***	0.55 (0.51 to 0.59)***
*Other/still studying*	8.5 (2704)	8.4 (871)	8.5 (1833)	0.74 (0.68 to 0.82)***	0.53 (0.47 to 0.59)***
*None*	17.6 (5626)	25.6 (2653)	13.8 (2973)	0.40 (0.37 to 0.43)***	0.26 (0.24 to 0.29)***
**Full time work %(n)**					
*Yes*	47.4 (14621)	44.9 (4644)	48.8 (10502)	Reference	Reference
*No*	52.6 (16181)	55.1 (5707)	51.2 (11024)	0.85 (0.81to 0.91)***	0.72 (0.67 to 0.76)***

Note:

*** significant difference *p*<0.001.

Table 6 shows the average AUDIT-C scores as a function of the various SES measures and the results of the regression analysis assessing whether SES was a significant predictor of consumption. Although all social-grades and incomes had lower AUDIT-C scores relative to the reference categories AB and >£40,000, the relationship was U-shaped. In contrast, the relationship with education was linear whereby AUDIT-C scores decreased with increasingly fewer qualifications. Lower AUDIT-C scores were also found among those not in full-time work. The findings were inconclusive as to whether or not an association was present with car and home ownership.

**Table 6:** Association between measures of socio-economic status and AUDIT-C scores (drinkers only n = 21539).

			Unadjusted			Adjusted for gender, age and ethnicity		
	M	SD	β	95%CI	*p*	β	95%CI	*p*
Social-grade								
AB	4.21	2.33	Reference			Reference		
C1	4.13	2.51	-0.08	-0.17 to 0.01	0.078	-0.19	-0.27 to -0.10	<0.001
C2	4.08	2.52	-0.13	-0.23 to -0.03	0.01	-0.28	-0.38 to -0.19	<0.001
D	3.81	2.65	-0.39	-0.51 to -0.28	<0.001	-0.52	-0.63 to -0.41	<0.001
E	4.04	2.85	-0.17	-0.31 to -0.03	0.014	-0.16	-0.29 to -0.03	0.013
Tenure								
Owns home	4.28	2.73	Reference			Reference		
Does not own home	3.99	2.4	0.3	0.23 to 0.37	<0.001	0.05	-0.02 to 0.12	0.188
Income								
£40,000 +	4.44	2.76	Reference			Reference		
£17,500 to £39,999	4.03	2.73	-0.4	-0.50 to -0.31	<0.001	-0.3	-0.39 to -0.20	<0.001
£9,500 to £17,499	3.68	2.77	-0.75	-0.87 to -0.64	<0.001	-0.51	-0.62 to -0.40	<0.001
< £11,499	4.03	3.01	-0.4	-0.52 to -0.28	<0.001	-0.2	-0.32 to -0.09	<0.001
Car								
Owns car	4.12	2.46	Reference			Reference		
Does not own car	4.07	2.56	-0.05	-0.12 to 0.02	0.163	-0.04	-0.11 to 0.03	0.240
Education								
University	4.1	2.31	Reference			Reference		
A-level and equivalent	4.54	2.7	-0.57	-0.68 to -0.46	<0.001	0.19	0.10 to 0.29	<0.001
GCSE/vocational	4.09	2.57	-0.01	-0.10 to 0.08	0.814	-0.08	-0.16 to 0.01	0.066
Other/still studying	3.94	2.44	-0.16	-0.29 to -0.03	0.019	-0.17	-0.30 to -0.05	0.006
None	3.53	2.55	-0.57	-0.68 to -0.46	<0.001	-0.32	-0.42 to -0.21	<0.001
Work								
Full time work	4.26	2.5	Reference			Reference		
Not in full time work	3.93	2.53	-0.33	-0.40 to -0.26	<0.001	0.2	0.13 to 0.28	<0.001

Tables 7 and 8 show the average AUDIT-Harm and AUDIT-Dependence scores as a function of the various SES measures; and the results of the regression analysis assessing whether SES is a significant predictor of alcohol-related harm and dependence. For income, education and social-grade, AUDIT-Harm scores were largest in the most disadvantaged groups (i.e. <£11,499, no qualifications and E), with a number of non-significant associations for less disadvantaged groups. Those who did not own their own their own home and were not in full time work also had higher AUDIT-Harm scores. The findings were inconclusive as to whether or not an association was present with car ownership.

**Table 7:** Association between measures of socio-economic status and Audit-Harm scores (drinkers only n = 21539).

			Unadjusted			Adjusted for gender, ethnicity and age			Adjusted for gender, ethnicity, age and Audit-C		
	M	SD	β	95%CI	*p*	β	95%CI	*p*	β	95%CI	*p*
**Social-grade**											
*AB*	0.45	1.3	Reference			Reference			Reference		
*C1*	0.57	1.49	0.12	0.06 to 0.17	<0.001	0.01	-0.05 to 0.06	0.826	0.05	<0.01 to 0.10	0.035
*C2*	0.49	1.42	0.03	-0.03 to 0.09	0.284	-0.06	-0.12 to <0.01	0.041	0.01	-0.04 to 0.06	0.75
*D*	0.52	1.44	0.07	<0.01 to 0.14	0.044	-0.06	-0.12 to 0.01	0.09	0.07	0.01 to 0.13	0.031
*E*	0.82	2.02	0.37	0.29 to 0.45	<0.001	0.29	0.21 to 0.37	<0.001	0.33	0.26 to 0.40	<0.001
**Tenure**											
*Owns home*	0.39	1.22	Reference			Reference			Reference		
*Does not own home*	0.82	1.84	0.42	0.38 to 0.47	<0.001	0.21	0.17 to 0.26	<0.001	0.2	0.16 to 0.24	<0.001
I**ncome**											
*£40*,*000 +*	0.52	1.55	Reference			Reference			Reference		
*£17*,*500 to £39*,*999*	0.47	1.46	-0.05	-0.10 to 0.01	0.076	-0.02	-0.07 to 0.04	0.527	0.05	<0.01 to 0.10	0.043
*£9*,*500 to £17*,*499*	0.45	1.53	-0.06	-0.13 to <0.01	0.056	-0.01	-0.07 to 0.06	0.867	0.12	0.05 to 0.18	<0.001
*< £11*,*499*	0.78	2.16	0.26	0.19 to 0.34	<0.001	0.27	0.20 to 0.34	<0.001	0.32	0.25 to 0.39	<0.001
**Car ownership**											
*Owns car*	0.48	1.37	Reference			Reference			Reference		
*Does not own car*	0.57	1.55	0.1	0.05 to 0.14	<0.001	0.06	0.02 to 0.10	0.006	0.02	-0.01 to 0.06	0.178
**Educational qualifications**											
*University*	0.49	1.34	Reference			Reference			Reference		
*A-level and equivalent*	0.77	1.76	0.28	0.22 to 0.33	<0.001	0.07	0.02 to 0.13	0.012	0.03	-0.03 to 0.08	0.311
*GCSE/vocational*	0.55	1.53	0.06	0.01 to 0.11	0.02	0.01	-0.04 to 0.06	0.734	0.03	-0.02 to 0.07	0.243
*Other/still studying*	0.42	1.27	-0.07	-0.15 to <0.01	0.056	-0.06	-0.14 to 0.01	0.096	-0.02	-0.09 to 0.05	0.526
*None*	0.35	1.31	-0.14	-0.20 to -0.07	<0.001	0.01	-0.06 to 0.08	0.765	0.09	0.03 to 0.15	0.005
**Employment status**											
*Full time work*	0.55	1.42	Reference			Reference			Reference		
*Not in full time work*	0.53	1.53	-0.02	-0.06 to 0.02	0.296	0.27	0.22 to 0.31	<0.001	0.22	0.18 to 0.26	<0.001

**Table 8:** Association between measures of socio-economic status and Audit-Dependence scores (drinkers only n = 21539).

			Unadjusted			Adjusted for gender and age			Adjusted for gender, age and Audit-C		
	M	SD	β	95%CI	*p*	β	95%CI	*p*	β	95%CI	*p*
**Social-grade**											
*AB*	0.13	0.61	Reference			Reference			Reference		
*C1*	0.19	0.72	0.06	0.03 to 0.09	<0.001	0.01	-0.02 to 0.04	0.39	0.03	<0.01 to 0.06	0.022
*C2*	0.22	0.86	0.09	0.06 to 0.12	<0.001	0.05	0.02 to 0.09	0.002	0.08	0.05 to 0.11	<0.001
*D*	0.22	0.87	0.09	0.05 to 0.13	<0.001	0.04	<0.01 to 0.08	0.066	0.09	0.06 to 0.13	<0.001
*E*	0.47	1.57	0.34	0.30 to 0.39	<0.001	0.31	0.26 to 0.36	<0.001	0.33	0.29 to 0.37	<0.001
**Tenure**											
*Owns home*	0.12	0.62	Reference			Reference			Reference		
*Does not own home*	0.37	1.17	0.24	0.22 to 0.27	<0.001	0.16	0.14 to 0.19	<0.001	0.16	0.13 to 0.18	<0.001
**Income**											
*£40*,*000 +*	0.18	0.89	Reference			Reference			Reference		
*£17*,*500 to £39*,*999*	0.16	0.88	-0.02	-0.06 to 0.02	0.275	<0.01	-0.04 to 0.03	0.876	0.03	-0.01 to 0.06	0.107
*£9*,*500 to £17*,*499*	0.2	0.96	0.02	-0.02 to 0.06	0.383	0.05	0.01 to 0.09	0.022	0.1	0.06 to 0.15	<0.001
*< £11*,*499*	0.36	1.34	0.18	0.14 to 0.22	<0.001	0.19	0.15 to 0.23	<0.001	0.21	0.17 to 0.25	<0.001
**Car**											
*Owns car*	0.15	0.68	Reference			Reference			Reference		
*Does not own car*	0.24	0.94	0.09	0.07 to 0.11	<0.001	0.07	0.05 to 0.10	<0.001	0.02	<0.01 to 0.04	0.048
**Education**											
*University*	0.16	0.69	Reference			Reference			Reference		
*A-level and equivalent*	0.29	0.94	0.13	0.10 to 0.17	<0.001	0.06	0.03 to 0.10	<0.001	0.04	0.01 to 0.07	0.013
*GCSE/vocational*	0.22	0.89	0.06	0.03 to 0.09	<0.001	0.05	0.02 to 0.08	0.001	0.06	0.03 to 0.09	<0.001
*Other/still studying*	0.18	0.75	0.02	-0.02 to 0.07	0.354	0.04	<0.01 to 0.09	0.061	0.06	0.02 to 0.10	0.004
*None*	0.2	1.03	0.04	0.01 to 0.08	0.025	0.12	0.09 to 0.16	<0.001	0.16	0.12 to 0.20	<0.001
**Employment status**											
*Full time work*	0.2	0.76	Reference			Reference			Reference		
*Not in full time work*	0.21	0.93	0.01	-0.01 to 0.04	0.245	0.15	0.13 to 0.18	<0.001	0.13	0.11 to 0.16	<0.001

Non-linear relationships were found between education and social-grade with AUDIT-Dependence scores. Although lower scores were found among all social-grades relative to AB and educational attainments relative to having a university qualification, similar scores were found amongst those of middle level SES, whilst the difference was greatest for those of the lowest SES. A linear association was noted with income, whereby decreasing income was associated with greater AUDIT-Dependence scores. Not owning a home or car and not being in full time work were also associated with greater dependency.

### Is the Alcohol Harm Paradox moderated by demographic characteristics?

Table 9 shows the results of the moderation analysis. The coefficient for the composite score can be interpreted as the effect on AUDIT dimension measures when age and gender are equal to zero i.e. for those aged 16–24 years of age and males, respectively. The decrease in AUDIT-C and increase in AUDIT-Harm with greater social-disadvantage, as measured by the composite score, did not appear to be moderated by gender. In contrast, increasing AUDIT-Dependence scores with increasing social-disadvantage was found to be greatest among men.

**Table 9:** Moderation effects of gender and age on the association between composite measures of socio-economic status and AUDIT dimensions.

	AUDIT-C			AUDIT-Harm			AUDIT-Dependence		
	β	95%CI	*p*	β	95%CI	*p*	β	95%CI	*p*
Intercept (Ref: male)	5.1	4.95 to 5.25	<0.001	0.47	0.38 to 0.53	<0.001	-0.03	-0.08 to 0.02	0.249
Gender	-1.02	-1.23 to -0.80	<0.001	-0.23	-0.36 to -0.10	0.001	0.03	-0.05 to 0.10	0.444
Composite score	-0.11	-0.14 to -0.08	<0.001	0.04	0.02 to 0.06	<0.001	0.06	0.05 to 0.07	<0.001
Gender x Composite score	<0.01	-0.05 to 0.04	0.834	<0.01	-0.03 to 0.03	0.983	-0.03	-0.04 to -0.01	0.001
Intercept (Ref: 16–24)	4.91	4.55 to 5.26	<0.001	1.02	0.8 to 1.24	<0.001	-0.02	-0.15 to 0.10	0.730
Aged 25–34	-0.7	-1.16 to -0.25	0.003	-0.65	-0.93 to -0.36	<0.001	0.04	-0.13 to 0.20	0.669
Aged 35–44	-0.57	-1.00 to -0.13	0.012	-0.84	-1.11 to -0.56	<0.001	-0.07	-0.22 to 0.09	0.408
Aged 45–54	-0.42	-0.85 to 0.01	0.055	-0.85	-1.11 to -0.59	<0.001	-0.04	-0.19 to 0.11	0.585
Aged 55–64	-0.25	-0.69 to 0.18	0.257	-0.75	-1.02 to -0.48	<0.001	-0.03	-0.18 to 0.12	0.716
Aged 65+	-0.23	-0.65 to 0.19	0.278	-0.74	-1.00 to -0.48	<0.001	0.01	-0.14 to 0.16	0.933
Composite score	-0.01	-0.07 to 0.06	0.854	0.05	<0.01 to 0.09	0.035	0.1	0.07 to 0.12	<0.001
Aged 25–34 x Composite score	<0.01	-0.09 to 0.09	0.971	0.01	-0.04 to 0.07	0.603	-0.04	-0.07 to <0.01	0.032
Aged 35–44 x Composite score	-0.02	-0.11 to 0.07	0.699	0.04	-0.01 to 0.10	0.137	-0.03	-0.06 to 0.01	0.103
Aged 45–54 x Composite score	-0.04	-0.13 to 0.04	0.336	0.03	-0.02 to 0.08	0.291	-0.04	-0.07 to -0.01	0.019
Aged 55–64 x Composite score	-0.11	-0.20 to -0.30	0.011	-0.03	-0.08 to 0.02	0.28	-0.06	-0.09 to -0.03	<0.001
Aged 65+ x Composite score	-0.25	-0.33 to -0.17	<0.001	-0.06	-0.11 to -0.01	0.011	-0.08	-0.11 to -0.06	<0.001

Note: Data were weighted to match the population in England; To correct for multiple comparisons the False Discovery Rate was applied (Benjamini and Yekutieli, 2001)

Interactions were also found between the three AUDIT dimensions and age. The findings were inconclusive as to whether or not social-disadvantage was associated with AUDIT-C scores among younger age groups. In contrast, those aged 65+ were found to have lower scores with increasing social-disadvantage. AUDIT-Harm scores increased with decreasing SES among all age groups, except among those aged 55–64 where no association was found and those and 65+ in which higher composite scores were associated with lower harm scores. Higher AUDIT-Dependence scores were found with increasing composite scores among all age groups, with the strongest association in younger age groups.

## Discussion

The Alcohol Harm Paradox emerged for a composite measure of SES. However, several SES indices showed additional non-monotonic associations with AUDIT-C, AUDIT-Harm and AUDIT-Dependence dimensions. The association between AUDIT-Dependence scores and lower SES was stronger for men. Lower SES was only associated with lower AUDIT-C scores among those aged 55+. Those aged 65+ also experienced less harm and dependence with decreasing SES than younger age groups.

Although these findings support the existence of the alcohol harm paradox, the presence of several monotonic relationships suggests that it may not work on a social gradient. Rather than running in a linear manner from top to bottom of the socioeconomic spectrum, it appears to be a phenomenon more of the very poorest. Previous studies have found wider differences in the proportion of individuals engaging in unhealthy behaviours between the bottom and top SES groups [54] and that alcohol-related harm is disproportionately experienced by the most deprived [55]. Although we adjusted for several demographic characteristics some of this non-linearity may be accounted for by other factors which are correlated with SES, including area level deprivation [56], access to treatment [4, 5] and marital status [57].

These results also suggest that the Alcohol Harm Paradox may be somewhat dependent on the measure of SES which is used. A number of studies have similarly failed to report strong associations between measures of consumption/harm and both educational qualifications and assets (e.g. home ownership and durables); while stronger relationships have been noted with income and employment status [15, 19, 31].

These findings have several implications. First they suggest that researchers should perhaps consider the measures of SES that have the most consistent linear relationship with measures of alcohol consumption, harm and possible dependence (i.e. income), if they wish to decipher the mechanisms of the Alcohol Harm Paradox. However, this approach may be an over-simplification as no single measure entirely captures the multifaceted nature of SES [43]; thus one may wish instead to use a composite score, which also has the advantage of reducing the effects of measurement error [21]. Secondly, these findings may explain the previous failure of alcohol control policies aimed at tackling health inequalities. For example, educational messages have had relatively little effect in reducing differences in consumption rates [58]. Although, this is also likely to be a consequence of the provision of information and persuasion to reduce alcohol related harm occurring in an environment in which many competing messages are received in the form of marketing and social norms supporting drinking. In contrast, interventions which tackle affordability appear to be more fruitful. Research indicates that minimum unit pricing (MUP) would have a positive impact on reducing health inequalities, by targeting price increases on heavier drinkers in the lower SES groups who are at greatest risk of harm [59]. Thirdly, these findings may help to elucidate the mechanisms of the Alcohol Harm Paradox. The association with income would suggest a materialistic explanation, while associations with occupational characteristics suggest possible psychosocial links between socioeconomic status and health. Thus it may be the case that those of lower SES experience greater harm as they have less access to health-care resources and have more deprived living conditions [4, 5].

Previous studies have established that increasing social-disadvantage is associated with severity of dependence among men [27, 28]. There are numerous possible reasons for this: women tend to have a greater number of emotional supportive social relationships, which may protect against the consequences of being unemployed, being on a low income, lack of educational qualifications, and excessive alcohol use [60]. Alternatively, it could be the result of the type of beverage which is consumed or differences in drinking patterns and drinking occasions; or the gendered meaning of drinking [26, 61–63].

The finding that the association between alcohol dependency and SES diminished in older age groups, whilst the association between consumption and SES increased, is also consistent with previous findings [24, 25]; and suggests that the Alcohol Harm Paradox may be a phenomenon particularly associated with the young. A number of explanations for the Alcohol Harm Paradox have been put forward which may elucidate this moderation effect. One theory is that differences exist in drinking patterns, whereby those from lower SES consume a similar amount of alcohol per week but do so over a smaller number of days. Indeed, “binge drinking” culture is a particularly associated with young adults and lower SES groups [64, 65]. There could also be a confounding effect due to poly-behaviours such a poor diet and lack of exercise [66]; or perhaps those living in more deprived areas face greater barriers to accessing health services and alcohol interventions [19]. Such poly behaviours and poor help-seeking have been shown to be more prevalent in younger age groups [67, 68]. The moderation effect of age may also be a methodological artefact. Older drinkers from lower SES groups could have been disproportionately excluded from the study as they are homeless or based in residential care/hospitals [69]. At the same time, research suggests that those from lower SES die from alcohol-related disorders at a younger age, and so older age groups may reflect the ‘healthier’ drinkers from lower SES groups [18].

This study has several strengths including its large sample size, use of multiple measures of SES and use of a validated measure of alcohol consumption and alcohol-related problems [45]. However, as with all cross-sectional surveys, caution should be taken when assigning cause and effect. It may be the case that SES has a direct influence on drinking behaviour or that drinking behaviour has an effect on SES measures. For example, those who experience greater alcohol problems may be more likely to become unemployed. Self-report measures are also susceptible to recall bias. This paper also makes the assumption that a measure of harmful alcohol use equates to actual alcohol-related harm. There is evidence to suggest that AUDIT scores are predictive of general health and disability, and that the AUDIT does as well as laboratory markers at predicting consumption [70]. The findings are also consistent with previous studies which have used objective clinical measures [19]. Another limitation is that moderation effects were only assessed using the composite score. However, this was found to be a reliable measure of SES and recognised the multifaceted nature of the phenomenon [21, 44]. Finally, although this paper assessed a wide range of SES measures which reflect those used previously [71]; the measures did not fully address the social capital aspect of SES [71, 72]. This is something which may require further consideration, as family and friend networks are associated with health outcomes [73].

In summary, we confirmed the generalisability of the Alcohol Harm Paradox across different measures of SES, but those different measures appear to influence whether it is observed as a linear trend across SES groups or a phenomenon associated particularly with the most disadvantaged. The paradox also appears to be more concentrated in men and younger age groups.

## Supporting Information

S1 Table Association between individual SES measures and the 10-items of the AUDIT. (PDF)
