# Supplementary material for: Deconstructing the Alcohol Harm Paradox: A Population Based Survey of Adults in England
Source: PLoS One. 2016 Sep 28;11(9):e0160666. doi: 10.1371/journal.pone.0160666 (PMC5040414; doi:10.1371/journal.pone.0160666)
Supplement: S1 Table — (PDF) [file pone.0160666.s001.pdf]

Supplementary Table 1: Association between individual SES measures and the 10-items of the AUDIT

	AUDIT 1	AUDIT 2	AUDIT 3	AUDIT 4	AUDIT 5
	β (95%CI) *p*				
**Social-grade**					
AB	Reference	Reference	Reference	Reference	Reference
C1	-0.29 (-0.33 to -0.26) <0.001	0.14 (0.10 to 0.18) <0.001	0.07 (0.04 to 0.11) <0.001	0.02 (0.01 to 0.04) 0.008	0.03 (0.01 to 0.04) <0.001
C2	-0.38 (-0.43 to -0.34) <0.001	0.20 (0.16 to 0.25) <0.001	0.05 (0.01 to 0.09) 0.024	0.04 (0.02 to 0.06) <0.001	0.03 (0.01 to 0.04) 0.001
D	-0.04 (-0.06 to -0.01) 0.001	0.23 (0.18 to 0.29) <0.001	0.01 (-0.04 to 0.06) 0.762	0.04 (0.02 to 0.06) <0.001	0.02 (0.01 to 0.04) 0.010
E	-0.6 (-0.65 to -0.54) <0.001	0.34 (0.27 to 0.40) <0.001	0.09 (0.03 to 0.15) 0.002	0.14 (0.11 to 0.16) <0.001	0.11 (0.09 to 0.13) <0.001
**Tenure**					
Owns home	Reference	Reference	Reference	Reference	Reference
Does not own home	-0.35 (-0.38 to -0.32) <0.001	0.40 (0.37 to 0.43) <0.001	0.25 (0.22 to 0.28) <0.001	0.11(0.09 to 0.12) <0.001	0.09 (0.08 to 0.10) <0.001
**Income**					
£40,000 +	Reference	Reference	Reference	Reference	Reference
£17,500 to £39,999	-0.2 (-0.24 to -0.16) <0.001	-0.05 (-0.09 to <0.01) 0.046	-0.15 (-0.20 to -0.11) <0.001	-0.01 (-0.02 to 0.01) 0.555	-0.02 (-0.03 to <0.01) 0.020
£9,500 to £17,499	-0.36 (-0.41 to -0.31) <0.001	-0.14 (-0.19 to -0.08) <0.001	-0.26 (-0.31 to -0.21) <0.001	0.01 (-0.01 to 0.03) 0.423	-0.01 (-0.02 to 0.01) 0.556
< £11,499	-0.37 (-0.42 to -0.32) <0.001	0.07 (0.01 to 0.12) 0.015	-0.10 (-0.15 to -0.05) <0.001	0.06 (0.04 to 0.09) <0.001	0.07 (0.05 to 0.08) <0.001

Supplementary Table 1 continued: Association between individual SES measures and the 10-items of the AUDIT

	AUDIT 6	AUDIT 7	AUDIT 8	AUDIT 9	AUDIT 10
	β (95%CI) *p*				
**Social-grade**					
AB	Reference	Reference	Reference	Reference	Reference
C1	0.01 (<0.01 to 0.02) 0.081	0.02 (0.01 to 0.04) 0.007	0.07 (0.05 to 0.08) <0.001	0.05 (0.03 to 0.08) <0.001	-0.03 (-0.05 to -0.01) 0.013
C2	0.02 (0.01 to 0.03) <0.001	<0.01 (-0.01 to 0.02) 0.682	0.04 (0.03 to 0.06) <0.001	0.02 (<0.01 to 0.05) 0.096	-0.04 (-0.06 to -0.01) <0.001
D	0.03 (0.02 to 0.04) <0.001	0.01 (-0.01 to 0.03) 0.495	0.07 (0.05 to 0.09) <0.001	0.01 (-0.02 to 0.04) 0.566	-0.01 (-0.04 to 0.01) 0.326
E	0.09 (0.08 to 0.11) <0.001	0.09 (0.06 to 0.11) <0.001	0.14 (0.12 to 0.16) <0.001	0.07 (0.03 to 0.10) <0.001	0.07 (0.04 to 0.11) <0.001
**Tenure**					
Owns home	Reference	Reference	Reference	Reference	Reference
Does not own home	0.05 (0.04 to 0.05) <0.001	0.09 (0.07 to 0.10) <0.001	0.14 (0.13 to 0.16) <0.001	0.15 (0.13 to 0.17) <0.001	0.05 (0.03 to 0.07) <0.001
**Income**					
£40,000 +	Reference	Reference	Reference	Reference	Reference
£17,500 to £39,999	<0.01 (-0.01 to 0.01) 0.373	-0.01 (-0.03 to 0.01) 0.200	<0.01 (-0.01 to 0.02) 0.697	-0.04 (-0.06 to -0.01) 0.002	<0.01 (-0.03 to 0.02) 0.827
£9,500 to £17,499	0.01 (<0.01 to 0.03) 0.018	-0.01 (-0.03 to 0.01) 0.168	0.01 (-0.02 to 0.03) 0.586	-0.03 (-0.06 to -0.01) 0.019	-0.02 (-0.05 to <0.01) 0.109
< £11,499	0.05 (0.03 to 0.06) <0.001	0.05 (0.03 to 0.07) <0.001	0.10 (0.07 to 0.12) <0.001	0.07 (0.04 to 0.10) <0.001	0.05 (0.03 to 0.08) <0.001

Supplementary Table 1 continued: Association between individual SES measures and the 10-items of the AUDIT

	AUDIT 1	AUDIT 2	AUDIT 3	AUDIT 4	AUDIT 5
	β (95%CI) *p*				
**Car**					
Owns car	Reference	Reference	Reference	Reference	Reference
Does not own car	-0.13 (-0.17 to -0.1)<br><0.001	0.08 (0.04 to 0.11)<br><0.001	0.01 (-0.02 to 0.04)<br>0.662	0.04 (0.02 to 0.05)<br><0.001	0.03 (0.02 to 0.04)<br><0.001
**Education**					
University	Reference	Reference	Reference	Reference	Reference
A-level and equivalent	-0.24 (-0.28 to -0.20)<br><0.001	0.44 (0.39 to 0.48)<br><0.001	0.24 (0.20 to 0.29)<br><0.001	0.04 (0.02 to 0.06)<br><0.001	0.07 (0.06 to 0.09)<br><0.001
GCSE/vocational	-0.30 (-0.34 to -0.26)<br><0.001	0.24 (0.20 to 0.28)<br><0.001	0.05 (0.01 to 0.09)<br>0.008	0.03 (0.02 to 0.05)<br><0.001	0.02 (0.01 to 0.03)<br>0.004
Other/still studying	-0.17 (-0.22 to -0.11)<br><0.001	0.08 (0.02 to 0.15)<br>0.007	-0.07 (-0.13 to -0.02)<br>0.011	<0.01 (-0.02 to 0.02)<br>0.991	0.01 (-0.01 to 0.02)<br>0.568
None	-0.02 (-0.05 to 0.01)<br>0.116	0.01 (-0.04 to 0.06)<br>0.800	-0.19 (-0.24 to -0.14)<br><0.001	0.01 (-0.01 to 0.03)<br>0.224	<0.01 (-0.01 to 0.02)<br>0.634
**Work**					
Full time work	Reference	Reference	Reference	Reference	Reference
Not in full time work	0.11 (0.08 to 0.14)<br><0.001	-0.21 (-0.25 to -0.18)<br><0.001	-0.22 (-0.25 to -0.20)<br><0.001	-0.01 (-0.02 to 0.01)<br>0.411	0.01 (<0.01 to 0.02)<br>0.122
**Composite**	-0.06 (-0.06 to -0.05)<br><0.001	0.02 (0.01 to 0.02)<br><0.001	-0.01 (-0.01 to -0.01)<br><0.001	0.01 (0.01 to 0.01)<br><0.001	0.01 (<0.01 to 0.02)<br><0.001

Supplementary Table 1 continued: Association between individual SES measures and the 10-items of the AUDIT

	AUDIT 6	AUDIT 7	AUDIT 8	AUDIT 9	AUDIT 10
			β (95%CI) *p*		
**Car**					
Owns car	Reference	Reference	Reference	Reference	Reference
Does not own car	0.02 (0.01 to 0.03)<br><0.001	0.03 (0.01 to 0.04)<br><0.001	0.04 (0.03 to 0.05)<br><0.001	<0.01 (-0.01 to 0.02)<br>0.651	0.02 (0.01 to 0.04)<br>0.007
**Education**					
University	Reference	Reference	Reference	Reference	Reference
A-level and equivalent	0.02 (0.01 to 0.03)<br><0.001	0.05 (0.03 to 0.06)<br><0.001	0.14 (0.12 to 0.16)<br><0.001	0.11 (0.08 to 0.13)<br><0.001	-0.02 (-0.04 to 0.01)<br>0.136
GCSE/vocational	0.01 (<0.01 to 0.02)<br>0.005	<0.01 (-0.02 to 0.01)<br>0.667	0.05 (0.03 to 0.06)<br><0.001	0.03 (<0.01 to 0.05)<br>0.039	-0.01 (-0.03 to 0.01)<br>0.416
Other/still studying	0.02 (<0.01 to 0.03)<br>0.018	-0.03 (-0.05 to -0.01)<br>0.005	0.01 (-0.01 to 0.03)<br>0.409	-0.03 (-0.07 to 0.01)<br>0.094	-0.02 (-0.05 to 0.01)<br>0.166
None	0.03 (0.02 to 0.04)<br><0001	-0.05 (-0.07 to -0.03)<br><0.001	<0.01 (-0.02 to 0.02)<br>0.961	-0.07 (-0.10 to -0.05)<br><0.001	-0.01 (-0.04 to 0.01)<br>0.296
**Work**					
Full time work	Reference	Reference	Reference	Reference	Reference
Not in full time work	0.01 (<0.01 to 0.02)<br>0.001	<0.01 (-0.01 to 0.01)<br>0.952	-0.01 (-0.02 to <0.01)<br>0.077	-0.04 (-0.06 to -0.02)<br><0.001	0.03 (0.01 to 0.05)<br><0.001
**Composite**	0.01 (<0.01 to 0.01)<br><0.001	<0.001 (<0.01 to <0.01)<br>0.096	0.01 (0.01 to 0.01)<br><0.001	<0.01 (<0.01 to 0.01)<br>0.921	<0.01 (<0.01 to <0.01)<br>0.029

Note: AUDIT 1 Frequency AUDIT 2 Quantity AUDIT 3 Binge-drinking; AUDIT 4 Not able to stop drinking AUDIT 5 Failed to do what normally do AUDIT 6 Drink in the morning AUDIT 7 Feelings of remorse and guilt AUDIT 8 Forgetting AUDIT 9 Injury AUDIT 10 Concerns from others
